# Effect of acceptance and commitment therapy for depressive disorders: a meta-analysis

**DOI:** 10.1186/s12991-023-00462-1

**Published:** 2023-09-07

**Authors:** Bing Zhao, Qian Wang, Liping Wang, Jie Chen, Tongtong Yin, Jingxuan Zhang, Xiaojing Cheng, Ruihua Hou

**Affiliations:** 1 School of Humanities and Social Sciences, Binzhou Medical University, Yantai, China; 2 Shandong Mental Health Centre, Shandong University, Jinan, China; 3 Clinical and Experimental Sciences, Faculty of Medicine, University of Southampton, Southampton, UK

**Keywords:** Acceptance and commitment therapy, Depressive disorders, Randomized controlled trial, Meta-analysis

## Abstract

**Objective:**

To systematically evaluate the effect of Acceptance and Commitment Therapy (ACT) on depressive disorders.

**Methods:**

The electronic databases of Web of Science Core Collection, Pubmed, EMBASE, Cochrane Library, PsycInfo, CNKI, Wanfang and Weipu were used to select relevant publications**.** Screening, data extraction, and quality assessment were undertaken following PRISMA guidelines for preferred reporting of systematic reviews and meta-analysis. RevMan5.4 was used for meta-analysis.

**Results:**

11 studies with a total of 962 patients were included. Random-effects model analysis showed that ACT could effectively reduce the level of depressive symptoms in patients with depressive disorders (SMD = − 1.05, 95% CI: − 1.43–− 0.66, P < 0.00001), improve psychological flexibility (MD = 4.84, 95% CI: 2.70–6.97, P < 0.00001), and have good maintenance effect (SMD = − 0.70, 95% CI: − 1.15–− 0.25, P = 0.002). All differences were statistically significant.

**Conclusions:**

ACT not only improves depressive symptoms and psychological flexibility, but also has a good maintenance effect, and it is particularly effective in Chinese patients. Large randomized controlled trials are needed to validate the findings from this meta-analysis.

**Supplementary Information:**

The online version contains supplementary material available at 10.1186/s12991-023-00462-1.

## Introduction

Depressive disorder is a common mental disorder, with significant and persistent low mood, lack of energy, and loss of interest as the main clinical manifestations [1]. Currently, the global annual prevalence of depressive disorder is approximately 6% [2], and the lifetime prevalence is approximately 18% [3]. It has been ranked as the third cause of the burden of disease worldwide by the World Health Organization in the "Global Burden of Disease Survey" that depressive disorders are the third largest disease burden in the world, and it has been projected to be first global disease burden by 2030 [4].

Acceptance and commitment therapy (ACT) is one of the third wave behaviour therapies, developed by an American psychology professor Steven C. Hayes in the 1990s, based on relational frame theory (RFT) [5], with interventions around psychological flexibility. This psychological intervention is based on empirical evidence to enhance psychological flexibility by adopting acceptance and mindfulness techniques, as well as commitment and coping strategies. According to RFT, human language and cognition rely on a relational framework, enabling individuals to comprehend their surroundings. However, this framework can also lead to emotional and cognitive distress, often accompanied by the tendency to avoid certain experiences [6, 7]. Patients with depressive disorders often suffer from decreased psychological flexibility due to avoidance of negative experiences, which aggravates depressive symptoms [8–11]. Currently, ACT is used in patients with depressive disorders to improve mood, stabilize emotions, and establish and realize self-esteem [12], and has shown a good maintenance effect [13].

There has been growing interest in using ACT as an effective intervention for patients with depressive disorders. However, there is a lack of research synthesizing currently available evidence to confirm its effectiveness comparing to other treatment interventions and its long-term effect which could offer guidance for its use in clinical practice. The current study aimed to systematically evaluate current evidence and conduct a meta-analysis to investigate its effectiveness for patients who met diagnostic criteria for depressive disorders based on the Diagnostic and statistical manual of mental disorders (DSM).

## Methods

The current review and meta-analysis were conducted following PRISMA guidelines for preferred reporting of systematic reviews and meta-analysis [14].

### Search strategy

The electronic databases of Web of Science Core Collection, Pubmed, EMBASE, Cochrane Library, PsycINFO, as well as CNKI, Wanfang and Weipu were used to select relevant publications before 25^th^ July 2022. Search terms were used for Acceptance and Commitment Therapy (acceptance and commitment therapy OR acceptance based OR acceptance-based), and depressive disorders (depressive disorder OR depression OR depressive). Studies published in both English and Chinese in peer-reviewed journals were searched. We also examined reference lists of appropriate articles in order to identify additional relevant studies. Two investigators (BZ and QW) independently assessed the literature included in the review. Any discrepancies were discussed, and a consensus was reached.

### Inclusion and exclusion criteria for study selection

The PICOS framework which focuses on the Population (P), Intervention (I), Comparison (C), Outcomes (O), and (S) study was used for study selection. Studies were included if they met the following criteria: (P) study participants met the DSM-IV diagnostic criteria for depressive disorders and participants did not have comorbid mental or medical conditions; (I): the active treatment group underwent ACT; (C) the control group were on the waiting list, or received treatment as usual, CBT or CT; (S): study designs were randomized controlled trials. Studies were excluded if they used ACT as an adjunctive treatment, or any of the above information was missing or not accessible.

### Data extraction

Data were extracted using a pre-piloted structured form. In addition to bibliographic information, extraction processes sought the following data: population, sample size, sex (female %), age (Mean or Median or Range), severity of depression, treatment intervention and duration, outcome measures, and follow-up period (see details in Table 1).

**Table 1:** Data extraction table of study characteristics

Study	Year	Country	Sample size T/C	Mean age ± SD T/C	%Female	Diagnosis	Format		Setting	Outcome indicator	Follow-up months
							T	C		depression	
A-Tjak et al.	2018	Netherlands	82 44/38	42.52 ± 12.21 40.45 ± 12.55	51.2	Major depressive disorder (DSM-IV)	ACT (Offline, individual)	CBT	Twenty times every one to two weeks for 45 to 55 min	HDRS-17	6
Chunxiao et al.	2022	China	182 95/87	21.83 ± 1.47 21.68 ± 1.19	52.7	Mild depression (BDI-II & semi-structured interview)	iACT (Online, individual)	WLC	Six weekly 30-min sessions	BDI-II	3
Ernst et al.	2011	Netherlands	93 49/44	48.84 ± 11.34 49.23 ± 10.07	81.7	Mild or moderate psychological distress (NS)	ACT (Offline)	WL	Eight weekly 2-h sessions	CES-D	3
Lappalainen et al.	2015	Finland	39 19/20	50.32 ± 12.54 53.40 ± 13.35	71.8	Mild depression (DSM-IV & structured interview)	iACT (Offline, individual)	WLC	Six weekly sessions	BDI-II	12 but no results
Mehdi et al.	2020	Iran	60 30/30	23.72 ± 4.18 25.18 ± 4.23	73.3	Moderate depression (DSM-V & semi-structured interview using SCID-5 and BDI-II)	ACT (offline, group)	TAU	Eight weekly 90-min group sessions	BDI-II	2
Shima et al.	2017	Iran	19 10/9	25.2 ± 4.2*	100	Moderate depression (DSM-IV & SCID-I/CV*)	ACT (offline, group)	CT	Twelve times twice a week	BDI-II	3
Wendy et al.	2016	Netherlands	169 82/87	45.15 ± 10.78 48.54 ± 12.63	82.2	Mild depression (CES-D & semi-structured interview using MINI and SDS)	ACT (online, individual)	WLC	nine times in twelve weeks	CES-D	6
Ren Zhihong	2012	China	182 95/87	21.83 ± 1.47 21.68 ± 1.19	52.7	At least mild depression (BDI and a semi-structed interview)	ACT (online, individual)	WL	Six weekly sessions	BDI-II	3
Situ Yuyi	2022	China	60 30/30	19.50 ± 4.1 20.00 ± 4.0	40	Depression (NS)	ACT + TAU (offline)	TAU	60 to 90 min four times a week	HAMD	None
Chen Juan et al.	2021	China	60 30/30	32.19 ± 7.31 32.23 ± 7.37	38.3	Mild depression (NS)	ACT + TAU (offline, group)	TAU	Six weekly 60-min sessions	SDS	None
Hayes et al.	2011	Australia	38 22/16	14.61 ± 3.1 15.49 ± 1.35	71.1	At least moderate depression (DSM-IV)	ACT (offline)	TAU		RADS-2	3

*DSM-IV/DSM-V* Diagnostic and Statistical Manual of Mental Disorders, *MINI* the Dutch version of the Mini International Neuropsychiatric interview, *SCID-I/CV* structured clinical interview for DSM-IV, *Axis I* clinical version, *BDI* Beck Depression Inventory, *NS* not specified

### Quality assessment

Two researchers independently evaluated the quality of the included studies according to the Cochrane Handbook 5.0.1 risk of bias assessment criteria. The content of the assessment includes random allocation method, allocation concealment, blinding of participants and researchers, blinding of outcome measurers, integrity of outcome data, selective reporting, and other sources of bias [15]. The evaluators make judgments of "low", "unclear" and "high" risk of bias for each of the above 7 items. All articles with low bias were graded A, some articles with low bias were graded B, and those without low bias were graded C. Two researchers independently evaluated the literature, any discrepancies were discussed within the research group until a consensus was reached. Eleven studies were assessed as B by two investigators (BZ and QW) independently, and Cohen’s Kappa coefficient was 1.

### Statistical analysis

RevMan 5.4 software was used to conduct meta-analysis on the included literature, including: (1) heterogeneity test: heterogeneity test was performed on multiple independent studies, and the I^2^ was calculated. If P > 0.05, there was no heterogeneity among multiple studies and the fixed effect model was used for analysis; if P ≤ 0.05, the heterogeneity of multiple studies was statistically significant, then the random effect model was selected, and sensitivity analysis or subgroup analysis was used to explore the source of heterogeneity and its effect; (2) Combined effect size (ES) was calculated and statistically tested; the outcome measures of this study were numerical variables; (3) Outcome measures were presented by weighted mean difference (MD) and its 95% confidence interval (95%CI). When different scales were used for the same outcome, the standardized mean difference (SMD) was used for analysis.

## Results

### Overview of the study selection

**Fig. 1:**
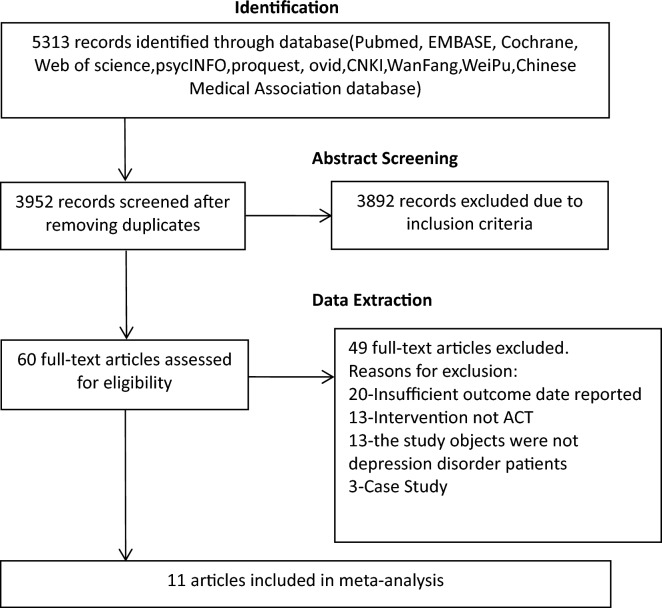
PRISMA flow chart

The current review and meta-analysis were conducted following PRISMA guidelines for preferred reporting of systematic reviews and meta-analysis and the PRISMA checklist (Additional file 1) [16]. The PRISMA flow chart (see Fig. 1) illustrates how studies were selected in this review and meta-analysis [17]. Our search strategy yielded an initial total of 5340 articles including 5163 articles in English and 177 articles in Chinese. 3952 potentially relevant articles remained after duplicates were removed. The titles and abstracts of these articles were then screened and 3892 articles were excluded. 60 full-length articles were then reviewed. Finally, 11 articles (involving 962 study participants) which met both inclusion and exclusion criteria were included.

### Study characteristics and intervention approaches

All 11 studies are randomised controlled trials, however, the forms of ACT delivery are diverse including 3 trials using group therapy [18–20], and 5 using individual therapy [21–25], and 3 trials [23, 26, 27] did not report specific delivery methods. ACT in 4 studies was delivered online [22–25]. Control groups included 5 studies [22–25, 27] on the waiting list, 4 studies receiving treatment as usual [18, 19, 26, 28], and one study [21] receiving CBT, and one study [20] receiving CT. In addition, 8 articles [19–25, 27] reported follow-up assessments between 2 and 12 months, see details in Table 1.

### Risk of bias assessment of the included studies

**Fig. 2:**
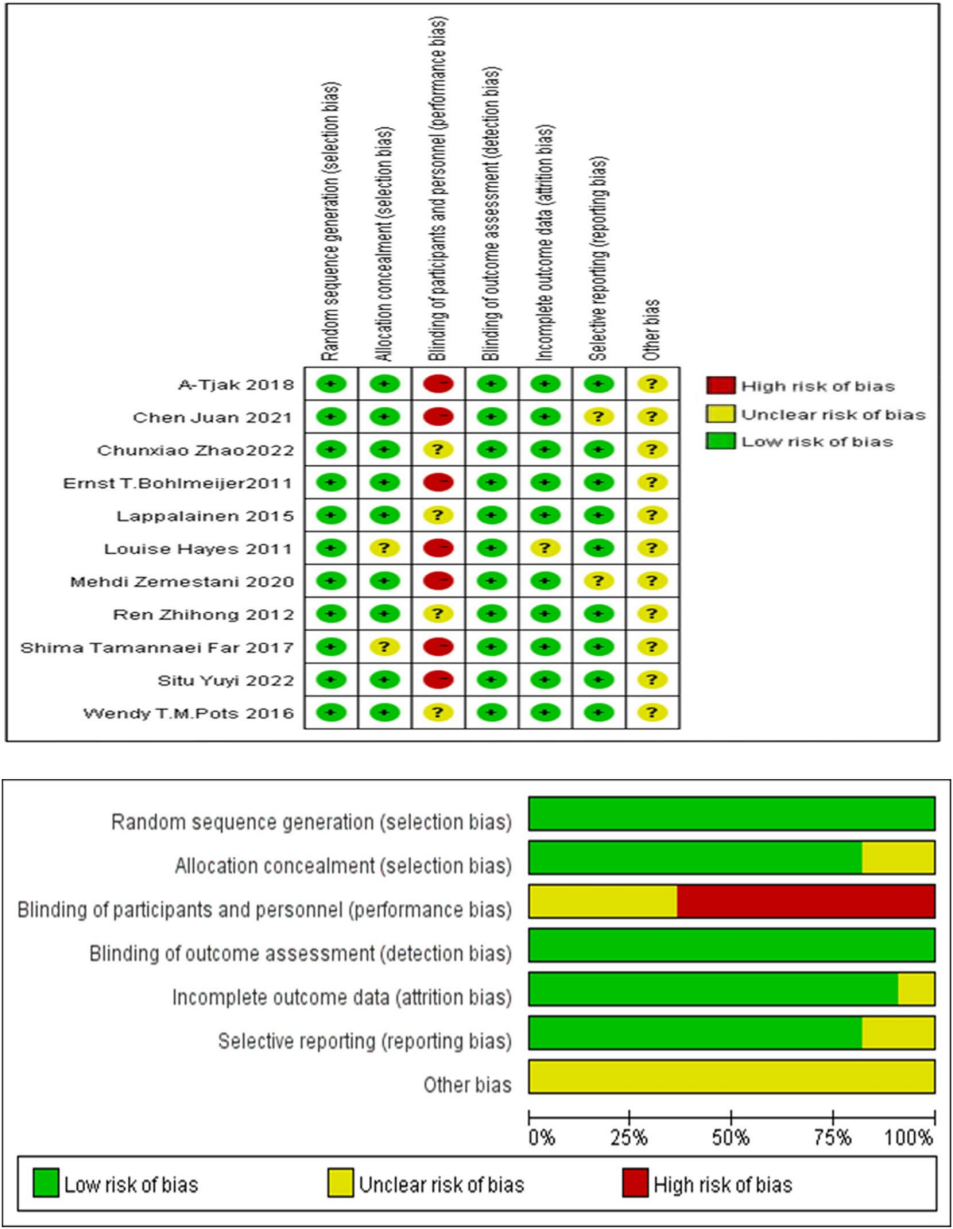
Risk of bias summary

**Fig. 3:**
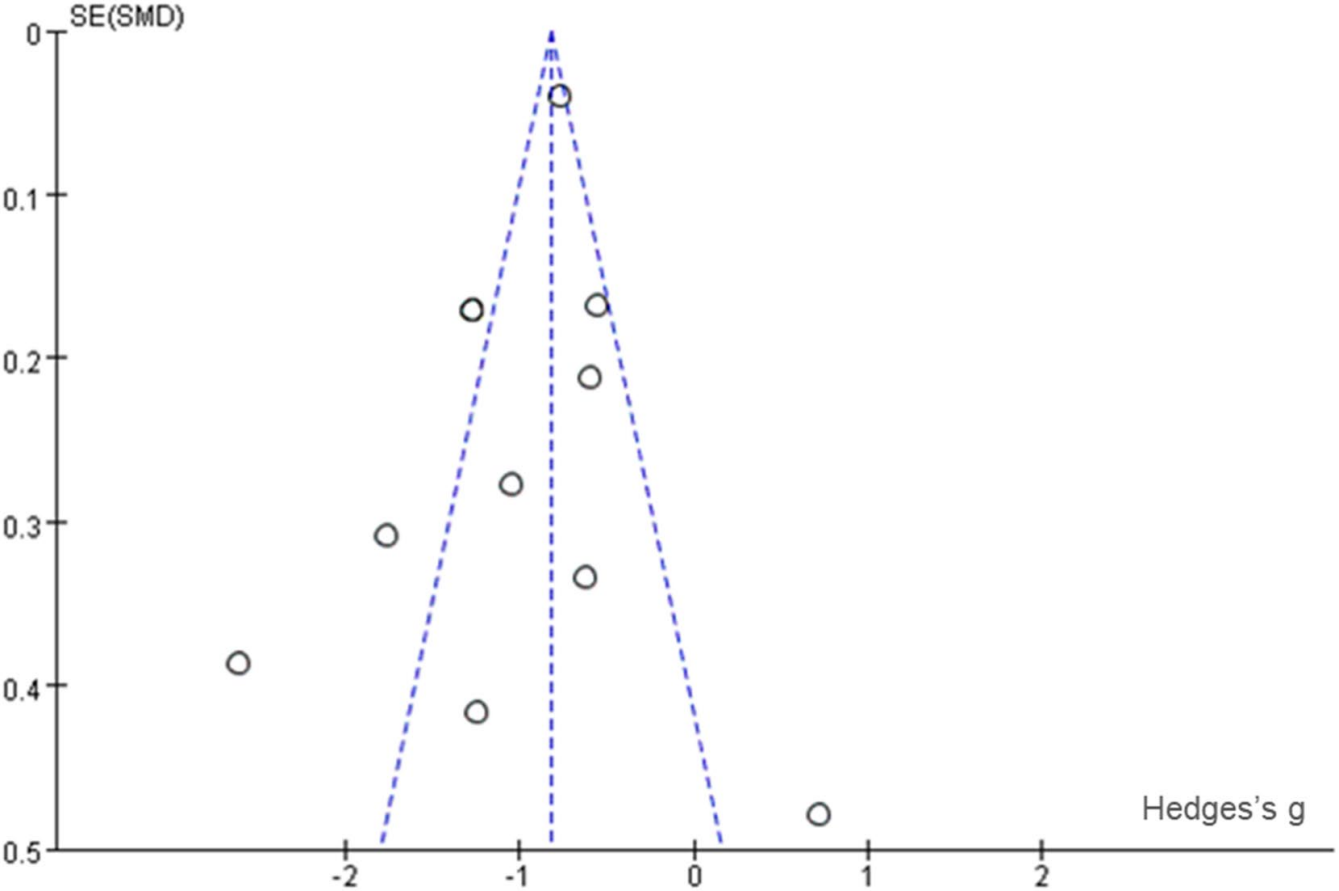
Funnel plot of standard error by Hedges’s g

As indicated in Fig. 2, there were of selection bias, reporting bias, detection bias, and attrition bias across the included studies. However, there was high risk of performance bias in terms of blinding of participants and researchers due to the nature of the ACT delivery. In addition, funnel plot of standard error by Hedges'g was used to indicate any potential publication bias. As shown in Fig. 3, the funnel plot showed marked asymmetry indicating heterogeneity amongst the studies which could be due to differences in outcome measures for depression, sample sizes, and analysis methods used across studies.

### Effect of ACT on depression

**Fig. 4:**
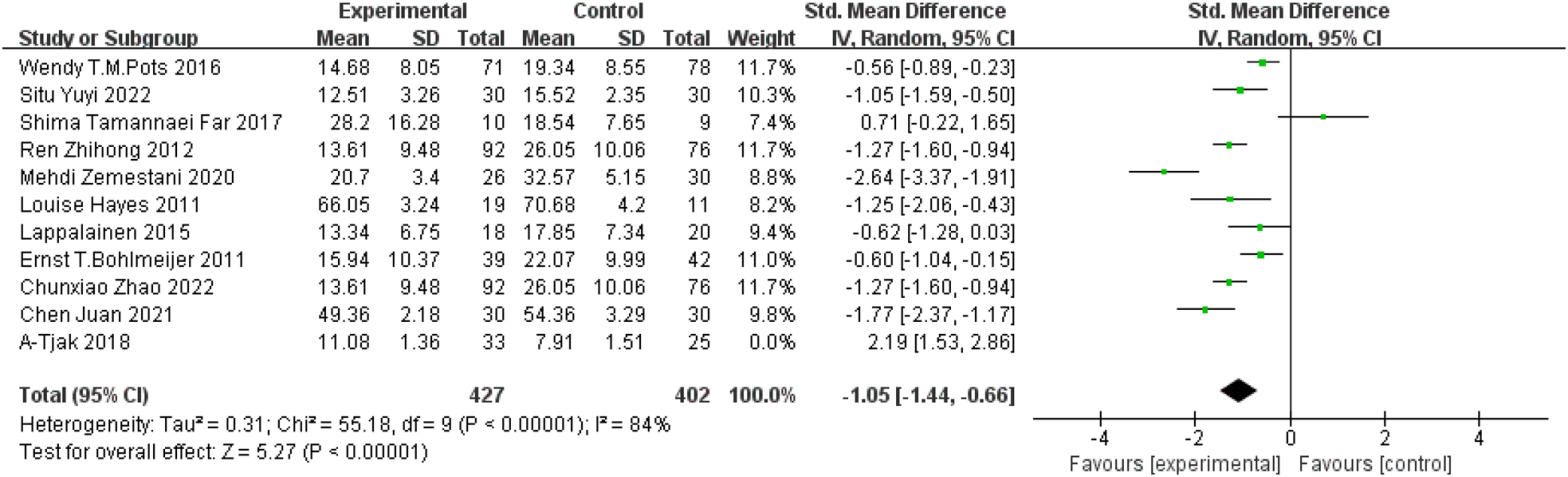
Effect of ACT on depression in patients with depressive disorders after sensitivity analysis

Eleven studies reported the effect of ACT on depression levels in 887 patients with depressive disorders. There was statistically significant heterogeneity among the studies (P < 0.00001; I^2^= 93%), therefore, the random effects model was used to conduct meta-analysis. There was significant treatment effect from ACT in comparison to other treatments, as shown in the forest plot in Fig. 4.

**Fig. 5:**
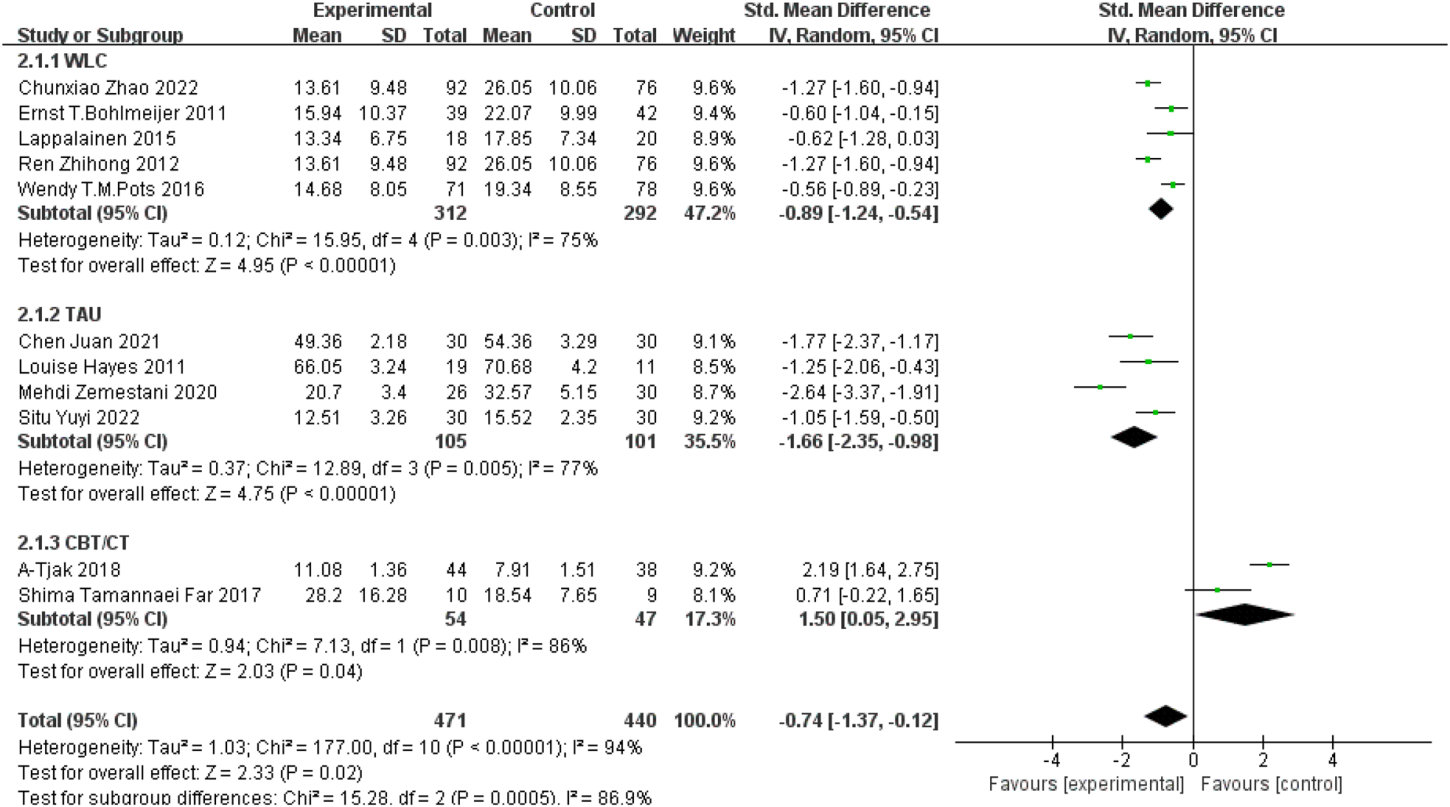
Subgroup analysis based on different control treatments

Subgroup analyses were conducted based on different control groups. Among 11 studies, there were 5 studies using patients on the waiting list as the control [22–25, 27] and meta-analysis revealed better treatment effect on depression from ACT (SMD = − 0.89, 95% CI: − 1.24–− 0.54, P < 0.00001). 4 studies used conventional treatment group as the control group [18, 19, 26, 28] and there were better treatment effects of ACT in improving depression (SMD = − 1.66, 95% CI: − 2.35–− 0.98, P < 0.00001). However, when compared to studies using CBT and CT as control groups [20, 21], treatment effect on depression favoured CBT and CT treatment group instead of ACT (SMD = 1.50, 95% *CI*: 0.05–2.95, *P* = 0.04). Results were indicated in forest plots in Fig. 5. The use of different control groups contributed to the high heterogeneity across studies (P = 0.0005; I^2^ = 86.9% > 50%).

**Fig. 6:**
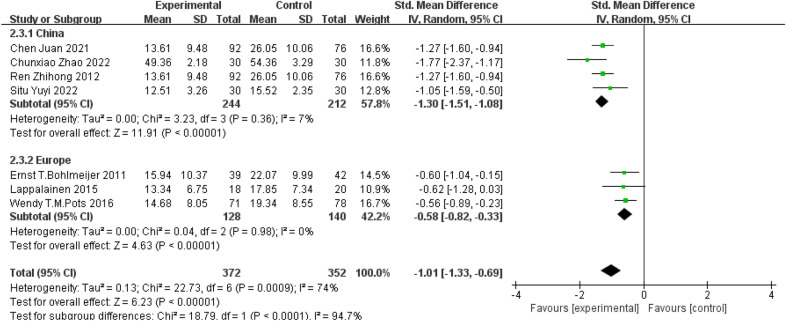
Subgroup analysis based on regions of study conducted

Subgroup analyses were also conducted based on regions of studies, as shown in forest plots in Fig. 6. When looking at 4 studies conducted in China using waiting list or conventional treatment as control [18, 22, 25, 26], there was no significant heterogeneity across studies (*P* = 0.36; *I*^*2*^= 7%) and the treatment effect on depression favoured ACT (SMD = -1.30, 95%CI: − 1.51–− 1.08, *P* < 0.00001). Similarly, when looking at 3 studies conducted in Europe [23, 24, 27], there was no significant heterogeneity across studies (*P* = 0.98; *I*^*2*^ = 0% < 50%) and the treatment effect on depression also favoured ACT (SMD = − 0.58, 95% *CI*: − 0.82–− 0.33, *P* < 0.00001).

**Fig. 7:**
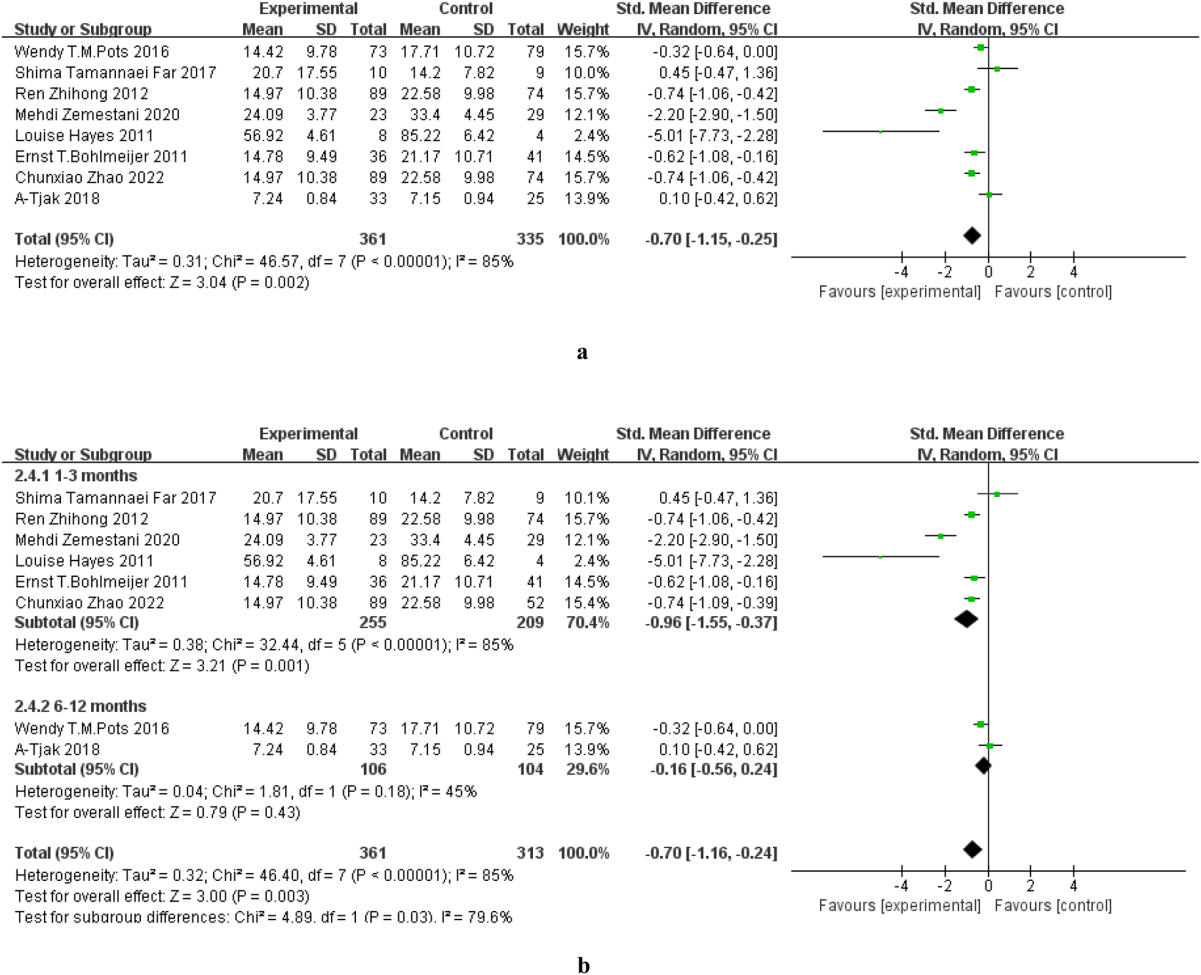
**a** Effect of ACT on depression during follow-up period. **b** Subgroup analysis based on follow-up duration

Eight studies [13, 14, 21–23, 26–28] completed follow-up assessments including 6 studies conducted within 3 months [14, 18, 21, 23, 27, 28] and 2 studies conducted after 6 months. While. the beneficial effects of ACT were seen in the follow-up period (SMD = − 0.70, 95% CI: − 1.15–− 0.25, P = 0.002), subgroup analyses revealed that the positive effect persisted at 3 months (SMD = -0.96, 95% CI: − 1.55–− 0.37, P = 0.001) but not after 6 (SMD = − 0.16, 95%CI: − 0.56–0.24, P = 0.43). The follow-up duration may contribute to the significant high heterogeneity across studies (P < 0.00001; I^2^ = 85%), see forest plots in Fig. 7.

### Effect of ACT on psychological flexibility

**Fig. 8:**
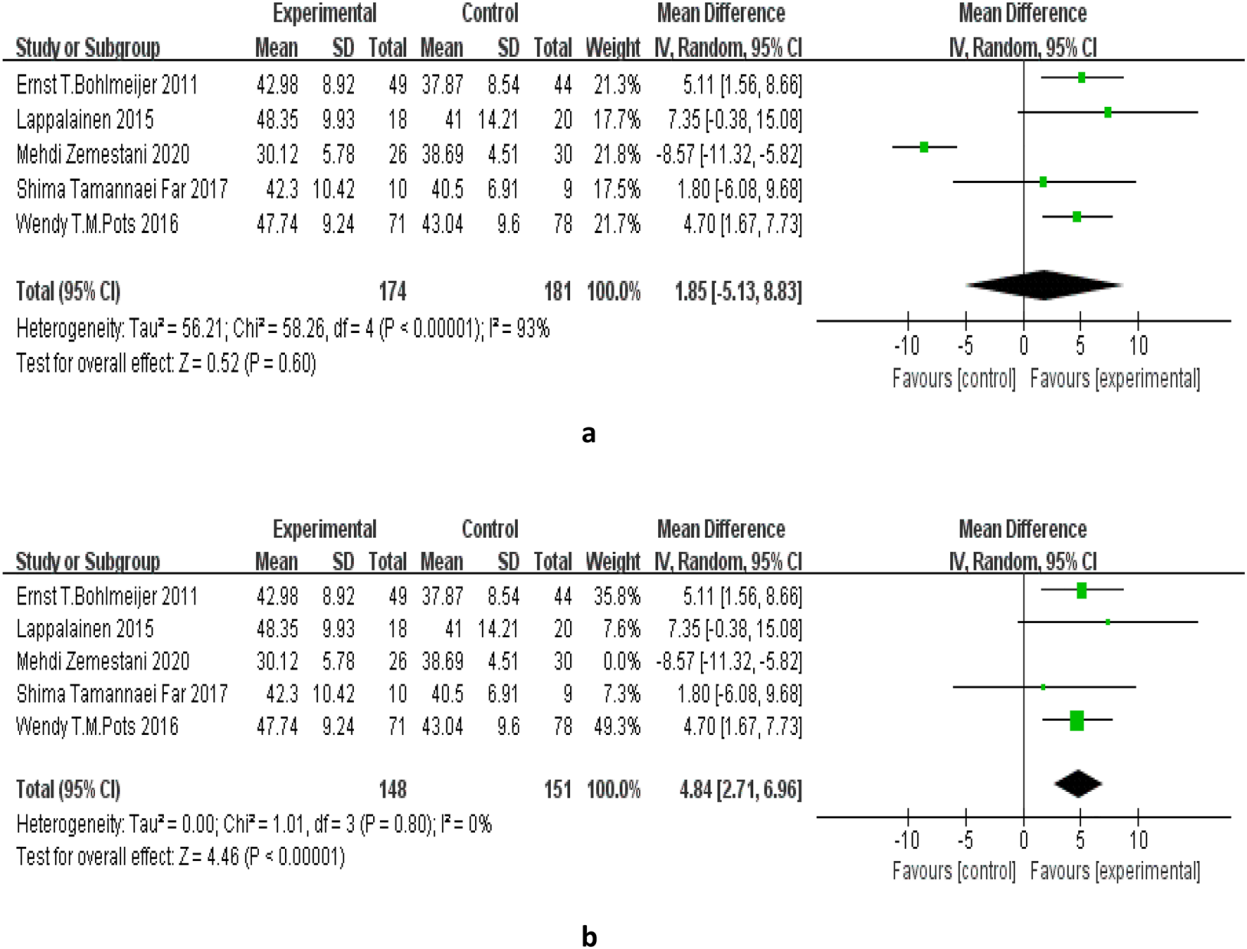
**a** Effects of ACT on psychological flexibility. **b** Effects of ACT on psychological flexibility after removing outlier

Among the 11 studies, 5 studies [19, 20, 23, 24, 27] reported changes in psychological flexibility after intervention, and the heterogeneity among the studies was statistically significant (P < 0.00001; I^2^ = 93%), meta-analysis using random effects model did not reveal any statistical difference between ACT and control treatment group (MD = 1.85, 95% CI: − 5.13–8.83, P = 0.60), see Fig. 8. It may be due to the study of Mehdi et al. [19], in which patients were depressive disorder patients with physical disabilities, resulting in large individual differences in the samples. Heterogeneity was not statistically significant after excluding this study (P = 0.80; I^2^ = 0%). In the remaining 4 studies [20, 23, 24, 27], compared with the control group, ACT showed significant effect on improving psychological flexibility (MD = 4.84, 95% CI: 2.71–6.96, P < 0.00001), see Fig. 8.

### Assessment of risk of bias

Summary assessments of the risk of bias were presented in Fig. 2, which included an assessment of 6 biases in each of those 11 studies. As indicated, there was a high-performance bias in 7 studies and there were low risks of selection bias, allocation, and attrition bias across studies. Each risk of bias item was also presented as percentages across all studies in Fig. 3. As shown in the funnel plot in Figure, the symmetrical distribution suggests that there was no publication bias across studies. And the Nfs,_0.05_ = 180.56 > 5 k + 10 = 65, indicate that the meta-analysis results are stable [29].

## Discussion

This study is the most up-to-date meta-analysis examining the effect of ACT for patients with depressive disorders. The findings highlight the beneficial effects of ACT on depression as well as its good maintenance effect 3 months after. The data also indicates that ACT can improve psychological flexibility which plays an important role in improving depression. Depression from an ACT perspective is seen as a consequence of psychological inflexibility, when individuals struggle with inner content such as thoughts and feelings which subsequently limits their ability to cope with emotional stress [30].

Research has found that depressed patients have low psychological flexibility which may contribute to their low mood [31]. ACT may improve depression by modulating psychological flexibility. Studies have shown that changes in cognitive content alone are not effective in preventing the recurrence of depression [32], what is more important is how individuals respond to stress[8]. When individuals are faced with complex life events, they normally have thoughts of avoidance, and avoidance behaviour. While avoidance seems to be able to help individuals solve the problem at the time, it is often avoidance behaviour that causes patients more distress, increase their depression, reduce their psychological flexibility, and gradually form a negative cycle in the long run. The focus of ACT is to improve psychological flexibility, through practices like acceptance and cognitive dissociation. Individuals are encouraged to acknowledge and embrace their thoughts and emotions, and learn to detach themselves from them [8, 33].

Subgroup analysis showed that ACT was effective in improving depression regardless of whether the control group was a waiting group or a treatment as usual group. However, the effect of ACT intervention was less effective than that of the CBT/CT group which is consistent with the findings of Ying Zhao et al. [34] who compared the efficacy of CBT to ACT on depression. One recent meta-analysis suggests that group ACT was found to be significantly superior to CBT in reducing depressive symptoms and the effect size can vary depending on the number of sessions provided and the primary condition of participants recruited [35]. The efficacy of these therapies is still controversial. The results of meta-analysis by Ruiz et al. [36] suggest that ACT is better than CBT for depressive disorders. A study conducted by Zhao et al. suggested that the beneficial effects of ACT compared with the CBT in college students with higher level of depression were mainly reflected in the long-term maintenance effect [33]. While ACT is a viable alternative to existing treatment regimens [37], more research is still needed to verify its effectiveness.

The current study also further examined the effect of ACT at different follow-up periods. The results showed that ACT had a good maintenance effect at 3 months compared with that of the control group, but its maintenance effect started to decrease afterwards, and there was no treatment effect at 6 and 12 month follow-up which is in line with a meta-analysis conducted by Bai et al. [38]. Evidence has also shown that ACT can maintain its treatment effect longer compared with CBT [32].

It is worth noting that the findings of this study suggest a better treatment effect of ACT in Chinese patients with depressive disorders in comparison to those of European patients. This may be due to the fact that ACT incorporates many concepts that are closely related to Eastern culture [39], such as acceptance and Taoism's flow with nature [40], as well as mindfulness techniques derived from Buddhist culture. Therefore, ACT is more in line with Eastern culture and values, or the Chinese community is more likely to accept the core ideas of ACT, so it has better acceptance [41] and better treatment effect in Chinese patient population. This suggests that ACT may be a potentially more effective intervention technique for Chinese patient population, so more research is needed to develop and tailor ACT that fits the Chinese context [39].

This review has certain limitations. Firstly, the limited number of studies meeting both inclusion and exclusion criteria, in particular, relatively small number of studies using a randomised controlled study design. Secondly outcome measures were mainly related to depressive symptoms and more research is needed to examine other behavioural changes and clinical outcomes, such as coping styles and quality of life. Thirdly, it should be noted that there was a high risk of performance bias due to difficulties in terms of blinding of participants and researchers [42]^.^ Lastly, the exclusion of grey literature may limit the comprehensiveness and timeliness of the available evidence.

## Conclusions

This study is the most up to date meta-analysis examining the effect of ACT for patients with depressive disorders. The findings highlight the beneficial effects of ACT on depression as well as its good maintenance effect 3 months after. The data also indicates that ACT can improve psychological flexibility which may act as a modulating factor in improving depression. In addition, the growing evidence from Chinese literature suggests its particular popularity in Chinese patient population.

### Supplementary Information

**Additional file 1.** PRISMA_2020_checklist.

## Data Availability

Datasets presented in this review will be accessed on request.
